# Non-invasive prediction of atrial cardiomyopathy characterized by multipolar high-density contact mapping

**DOI:** 10.1007/s10840-025-02001-2

**Published:** 2025-02-03

**Authors:** Moritz T. Huttelmaier, Alexander Gabel, Jonas Herting, Manuel Vogel, Stefan Störk, Stefan Frantz, Caroline Morbach, Thomas H. Fischer

**Affiliations:** 1 Dept. of Internal Medicine I, University Hospital Würzburg (UKW), University of Wuerzburg–University Clinic, Oberdürrbacherstr. 6, 97080 Würzburg, Germany; 2 Institute of Medical Virology, Goethe-University Frankfurt, 60596 Frankfurt am Main, Germany; 3 Infection Control and Antimicrobial Stewardship Unit, UKW, Würzburg, Germany; 4 Dept. Clinical Research & Epidemiology, Comprehensive Heart Failure Centre Würzburg, University Hospital Würzburg, Würzburg, Germany

**Keywords:** Atrial fibrillation, Atrial cardiomyopathy, Multipolar electroanatomical mapping, Echocardiography, Left atrial strain, Unsupervised machine learning

## Abstract

**Introduction:**

Atrial cardiomyopathy (AC) establishes links between atrial fibrillation (AF), left atrial (LA) mechanical dysfunction, structural remodeling, and thromboembolic events. Early diagnosis of AC may impact AF treatment and stroke risk prevention. Modern endocardial contact-mapping provides high-resolution electro-anatomical (EA) maps of the LA, thus allowing to display the myocardial substrate based on impaired signal amplitude and to characterize AC. Correlation of invasively assessed AC using a novel, multipolar mapping catheter (OCTARAY™, Biosense Webster, limited market release) and LA echocardiographic parameters could form the basis for a set of echo parameters for non-invasive prediction of AC.

**Methods:**

We retrospectively identified 50 adult patients who underwent primary pulmonary vein isolation (PVI) for paroxysmal or persistent AF between 08/22 and 05/23 fulfilling the selection criteria: (i) EA mapping with a novel multipolar mapping catheter (Octaray®); (ii) acquisition of voltage maps in sinus rhythm (SR) with ≥ 5000 points/map; and (iii) transthoracic echocardiography acquired in SR ≤ 48 h before PVI. Exclusion criterion was previous LA ablation. We generated EA maps with two sets of upper voltage thresholds (0.2–0.5 mV and 0.2–1.0 mV) and assessed total LA low voltage area (LVA). As LVA thresholds for the classification of AC are not yet established, an unsupervised machine learning cluster analysis was performed using a Gaussian mixture model (GMM), and two groups of patients with mild and severe AC were identified. Based on these two groups, we selected echo parameters for further analysis by applying the Boruta algorithm. The predictive capacity of the selected parameters was evaluated using a support vector machine.

**Results:**

The mean age of the studied sample (*n* = 50) was 63 ± 11 years, 62% were men, 64% showed persistent AF, median CHA_2_DS_2_-VASc score was 2 (quartiles 1, 3), and NT-proBNP was 190 (71, 391) pg/ml. A median of 5771 (5217, 6988) points/map were acquired. GMM yielded clusters of mild AC (*n* = 28) and severe AC (*n* = 22). Median LVA was 0.6 cm^2^ (< 0.5 mV) resp. 4.1 cm^2^ (< 1.0 mV) in group mild AC and 6.9 cm^2^ (< 0.5 mV) resp. 27.2 cm^2^ (< 1.0 mV) in group severe AC. Several echocardiographic parameters differed between the groups of mild and severe AC: dynamic LA parameters (end diastolic LA reservoir strain: 24.5% (22, 29) vs 15% (12, 19), *p* < 0.001; LA reservoir strain at atrial contraction: 22% (19, 25) vs 15% (11, 18), *p* < 0.001, end diastolic LA contraction strain: 13% (8, 15) vs 7.5% (3, 13), *p* < 0.01) as well as LA end-systolic volume index to a´ ratio (LAVI/*a*′: 297 (231,365) vs 510 (326,781), *p* < 0.01). Consistent distribution of NT-proBNP (mild AC: 125 (48,189) pg/ml, severe AC: 408 (254,557) pg/ml, *p* < 0.0001) and CHA_2_DS_2_-VASc score (mild AC: 1 (1–2), severe AC: 3 (3–4), *p* < 0.0001) served as proof of concept. Applying the selected echocardiographic parameters, the machine learning algorithm correctly identified both subgroups with a mean AUC of 0.9 (95% CI 0.83–0.99). At 12 months, AF recurrence rate was 10.7% in mild AC and 40.9% in severe AC (*p* < 0.05).

**Conclusion:**

Among patients qualifying for PVI, machine learning analysis of high-resolution LA maps allowed to identify subgroups with mild and severe AC avoiding the use of arbitrary LVA thresholds. The subgroups were predicted non-invasively with good accuracy using a machine learning approach that incorporated a set of echocardiographic markers. This data could advance the clinical triage of patients with AF.

**Graphical abstract:**

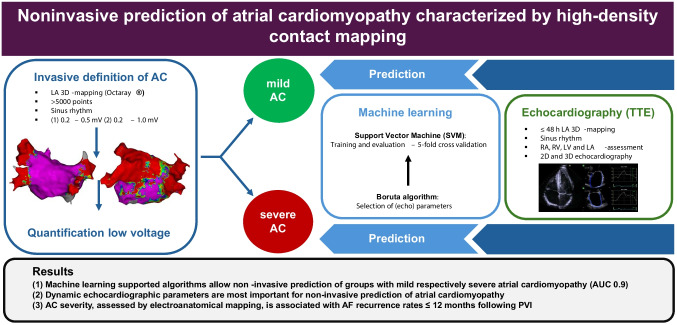

**Supplementary Information:**

The online version contains supplementary material available at 10.1007/s10840-025-02001-2.

## Introduction

Atrial fibrillation (AF) is a highly prevalent arrhythmia, which carries a markedly increased risk of thromboembolism and stroke [1]. The concept of “atrial cardiomyopathy (AC)” comprises different degenerative processes including electric instability (AF), left atrial (LA) mechanical dysfunction, and structural remodeling and scarring [2, 3]. Furthermore, AC is considered prothrombotic, which may explain thromboembolic events in patients with and without a history of AF. Therefore, early diagnosis of AC is of clinical importance as this may impact AF treatment and stroke risk prevention. However, up to date, robust and feasible methods for non-invasive diagnosis of AC are still a challenge in clinical practice, and no defined echocardiography-derived marker exists for the diagnosis of AC. Due to the limitation in spatial resolution, currently available cardiac MRI in clinical use is not sufficient for diagnosing and characterizing AC [4–6]. Modern endocardial contact-mapping provides high-resolution electro-anatomical maps of the LA, which holds promise to better display and quantify the myocardial substrate based on impaired signal amplitude and to invasively characterize the severity of AC [7–9]. Several studies in the field already performed correlations between invasively assessed low voltage areas (LVA) and LA function [9, 10]. The resolution of these maps, however, was relatively low, a non-evidence-based threshold of < 0.5 mV was chosen to demarcate LVA, and arbitrary LVA-cutoffs were used to define AC. The aim of this study was to take advantage of (i) improvements of invasive mapping technologies creating high-resolution substrate maps and (ii) established machine-learning approaches for operationalization of AC to avoid arbitrarily defined cutoff values. The correlation of invasively assessed AC using a novel, multipolar high-resolution mapping catheter (OCTARAY™, Biosense Webster, limited market release) and LA echocardiographic parameters could form the basis of an echocardiographic parameter set for non-invasive characterization and prediction of AC. Using a combination of unsupervised and supervised machine learning approaches, we aim to define an echocardiography-derived parameter set for characterization and prediction of LA AC defined by invasive quantification of LA LVA.

## Methods

### Study sample

We retrospectively identified 50 adult patients, who had undergone primary pulmonary vein isolation (PVI) for paroxysmal or persistent AF between 08/22 and 05/23 at the University Hospital Würzburg. All consecutive patients who underwent primary PVI using the CARTO® electroanatomical mapping system during the study period were considered. The following inclusion criteria applied: (i) Electro-anatomical mapping using a novel multipolar mapping catheter (OCTARAY™, Biosense Webster, Irvine, CA, USA); (ii) acquisition of voltage maps in sinus rhythm (SR) with ≥ 5000 points/map; (iii) transthoracic echocardiography (TTE) acquired in SR ≤ 48 h prior to PVI. Exclusion criteria were previous LA catheter ablation and/or previous LA cardiac surgery. The study was performed in line with the principles of the Declaration of Helsinki, and its later amendments and approval were granted by the Ethics Committee of the University Hospital Würzburg (reference number: 20230315 02).

### Clinical measurements and outcome ascertainment

**Fig. 1:**
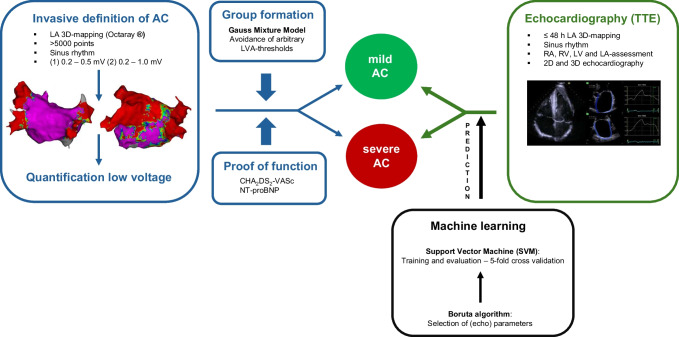
Visualization of the methodological design for non-invasive prediction of invasively assessed AC using an unsupervised machine learning approach

Figure 1 summarizes and illustrates our methodological approach analyzing electroanatomical maps and LA echocardiographic parameters with an unsupervised machine learning approach.

#### Echocardiography

Standardized transthoracic (TTE) echocardiography examinations (GE ultrasound system E95, probe 4Vc-D, GE Healthcare, Solingen, Germany) were performed by an experienced physician in accordance with current guidelines [11]. All off-line analyses were performed using the software ViewPoint 6 (ViewPoint 6 version 6.14, GE Healthcare, Solingen, Germany). We characterized left ventricular (LV) systolic function by LV ejection fraction (LVEF, Simpson’s method) and LV global longitudinal strain (GLS, two-dimensional (2D) speckle tracking). Right ventricular (RV) systolic function was assessed by tricuspid annular plane systolic excursion (TAPSE), fractional area change (FAC), and RV strain analysis (GLS), when applicable. We further assessed and graded LV diastolic function following current guidelines into grade 1–3 diastolic dysfunction based on LA volume, TR *V*_max_, *E*, and *e*´ values [12]. Right atrial (RA) systolic area was measured in an RV-focused four-chamber view. Minimal and maximal 2D LA volumes were measured in apical two- and four-chamber views, and minimal and maximal three-dimensional (3D) LA volumes were quantified. Based on LA volumes, we calculated 2D and 3D LA end-systolic volume index (LAVI), 2D and 3D LAVI/LV tissue Doppler relaxation velocity during atrial contraction (LAVI/*a*′), 2D and 3D total LAEF, 2D and 3D active LAEF, and 3D passive LAEF. Longitudinal atrial strain representing LA reservoir, LA conduit and LA contraction were analyzed semi-automatically off-line in two- and four-chamber views (2D) as well as based on a LA 3D dataset (3D) [13]. The zero-strain reference was set at the ventricular end-diastole (ed) and at the onset of atrial contraction (ac) in individual measurements.

#### Electro-anatomical mapping and catheter ablation

All electro-anatomical maps were acquired by two experienced interventional electrophysiologists with the same type of multipolar mapping catheter (OCTARAY™ 2–2-2–2-2, Biosense Webster, Irvine, CA, USA). This catheter provides 8 splines with a total of 48 electrodes with fixed inter-electrode spacing of 2 mm covering a surface area of 0.9 mm^2^/electrode. The CARTO® 3 system version 7 was used for 3D-anatomical mapping. We generated electroanatomical maps in SR with two different upper voltage thresholds (0.2–0.5 mV and 0.2–1.0 mV). Wide circumferential PVI was performed using a contact force-enabled irrigated tip radiofrequency (RF) ablation catheter (Smart Touch Thermocool, tip electrode: 3.5 mm, Biosense Webster, Irvine, CA, USA) aiming for an ablation index (AI) of 350 at the posterior LA and an AI of 450 at the anterior LA and the carinas. Additional ablation lines were applied if LA flutter occurred during the procedure or if atypical LA flutter was previously documented, and LVAs were present as a potential source of re-entry.

#### Operationalization of AC

We assessed LA LVA in all patients applying two voltage thresholds (0.2–0.5 mV and 0.2–1.0 mV). For this purpose, we separated the LA electro-anatomical map into five anatomically defined sections (anterior, posterior, lateral wall, bottom, and septum). The pulmonary veins (PVs) and the LA appendage (LAA) were excluded from further analysis. In every section, LVAs were manually traced for both voltage thresholds by a trained cardiac electrophysiologist certified by the European Heart Rhythm Association (EHRA). Group formation into groups with *mild AC* and *severe AC* were based on (i) the assumption of healthier and sicker individuals within the study cohort and (ii) the extent of LVA that correlates with the severity of AC. LVA-dependent cluster-analysis was performed using a Gaussian mixture model (GMM), setting the number of clusters to two based on the maximum score of the Bayesian information criterion for different numbers of clusters.

#### Laboratory testing

Laboratory analyses, performed routinely the day before catheter ablation, were retrieved from the hospital’s documentation system. In all patients, levels of N-terminal pro-B-type natriuretic peptide (NT-proBNP), creatinine, C-reactive protein (CRP), and HbA1c were assessed.

#### Outcome ascertainment

All patients were followed up routinely in our outpatient clinic 3 months after PVI by a trained cardiologist. Structured clinical assessment consisted of (i) physical examination, (ii) patient history with a focus on heart failure (HF) and symptomatic AF recurrence, (iii) 12-lead surface ECG, and (iv) assessment of current medication. Holter-ECGs performed on an outpatient basis were collected, evaluated, and stored digitally in the hospital’s documentation system. Twelve to 20 months after primary PVI, all patients were re-contacted for a structured telephone interview, the hospital’s documentation system was systematically searched for AF recurrences, and any resting- and Holter-ECGs performed in practices and/or external hospitals were collected.

### Data analysis

Statistical analyses and machine learning approaches were implemented with the programming language R, version 4.3.2. (R Foundation for Statistical Computing, Vienna, Austria). We described data using mean (standard deviation, SD), median (quartiles), or count (percent), as appropriate. Given the limited number of subjects and the assumption of healthier and sicker individuals within the study cohort, LVA-dependent cluster analysis was performed using a GMM, setting the number of clusters to two. Parameter training was based on running the expectation maximization (EM) algorithm 100 times, and parameter sets showing the maximum log-likelihood were used as model parameters. Because GMM is a soft clustering algorithm, the maximum of estimated cluster probabilities was used to assign each data point to a particular cluster, resp. subgroup. The selection of voltage thresholds for measuring LVAs (0.2–0.5 mV and 0.2–1.0 mV) was based on testing if at least one cluster within one voltage threshold could pass the Shapiro–Wilk test on normality. We used echocardiography-independent features such as CHA_2_DS_2_-VASc score and NT-proBNP levels to verify the plausibility of correct group allocation. In order to reduce the set of echocardiographic parameters and thereby increase the power to discriminate between the two subgroups, we applied the Boruta algorithm [14]. As the Boruta algorithm depends on a data set without missing data, we imputed missing data using the Amelia algorithm [15]. To assess the predictive capacity of the selected parameters, a support vector machine (SVM) with a linear kernel was trained. Using the classification and regression training package (caret) [16], the SVM was trained and further evaluated by a fivefold cross-validation. The performance of the SVM classifier was measured with the area under the receiving operator curve (AUROC) and its 95% confidence intervals (CI) by using the yardstick package [17]. Furthermore, to highlight statistical differences of each selected echocardiographic parameter between the two subgroups, we conducted a two-sided Mann–Whitney *U* test. To correct for multiple testing, the Benjamini-Yekutieli method [18] was performed, and adjusted *p* values below 0.05 were considered as statistically significant. The anonymised dataset as well as the detailed specification of the statistical analyses are available online (https://github.com/AlexGa/Noninvasive-prediction-of-atrial-cardiomyopathy).

## Results

### Baseline characteristics

All consecutive patients (*n* = 60) who underwent primary PVI using the CARTO® electroanatomical mapping system during the study period were considered. A total of *n* = 10 patients were excluded, predominantly because the TTE was not acquired in sinus rhythm prior to PVI. The mean age of the studied sample (*n* = 50) was 63 (± 11) years, 62% were men, and 64% showed persistent AF. The median CHA_2_DS_2_-VASc score was 2 (quartiles 1, 3), and the median NT-proBNP level was 190 (71, 391) pg/ml. In the studied sample, a median of 5771 (5217, 6988) points/map were acquired, and the median LVA (at voltage threshold 0.2–1.0 mV) was 7.8 (3.9, 22.9) cm^2^. The baseline characteristics of the total sample are further detailed in Table 1.

**Table 1:** Baseline characteristics of the total study sample

	Total sample *n* = 50
Age (years)	64 (56–70)
Male sex, % (*n*)	62 (31)
Body mass index (kg/m^2^)	28 (24.6–30.8)
**Atrial fibrillation**	
CHA_2_DS_2_-VASc score	2 (1–3)
Paroxysmal AF, % (*n*)	36 (18)
Persistent AF, % (*n*)	64 (32)
Antiarrhythmic medication, % (*n*)	38 (19)
**Cardiovascular risk factors**	
Arterial hypertension, % (*n*)	78 (39)
Previous stroke or TIA, % (n)	4 (2)
Diabetes mellitus, % (*n*)	14 (7)
**Electroanatomical mapping**	
Mapping points per map, *n*	5711 (5217–6988)
LVA cm^2^ (< 1.0 mV)	7.8 (3.9–22.9)
LVA cm^2^ (< 0.5 mV)	1.8 (0.5–5.9)
**Laboratory values**	
CRP (mg/dl)	0.1 (0.1–0.2)
eGFR (mg/ml/1.73 m^2^)	75 (63–85)
NT-proBNP (pg/ml)	189.5 (70.8–391.2)
HbA1c (%)	5.7 (5.4–6)
**Echocardiography**	
LVEF biplan (%)	61 (56–63)
GLPS 3D (-%)	17.6 (18.9–16.2)
LAVI 2D (ml/m^2^)	733 (524–890); *n* = 45
* E*/*e*′	7.6 (6–9); *n* = 49
TR Vmax (m/s)	2.5 (2.4–2.7); *n* = 31
**Outcome**	
AF recurrence ≤ 12 months, % (*n*)	24 (12)

Linear trait values are presented as median with interquartile range (25th–75th percentile). Relative and absolute frequencies of categorical variables are presented

*BMI* body mass index, *AF* atrial fibrillation, *LVA* low voltage area, *CRP* C-reactive protein, *eGFR* estimated glomerular filtration rate, *NT-proBNP* N-terminal pro-B-type natriuretic peptide, *LVEF* left ventricular ejection fraction, *GLPS* global longitudinal strain, *LAVI* left atrial volume index, *TR Vmax* maximal velocity of tricuspid regurgitation

### Cluster analysis: group mild and severe AC

The univariate voltage threshold < 1.0 mV and the measured LVA were revealed as most appropriate for cluster analysis due to the number of detected clusters and the robust estimation of parameters. The detailed results of the underlying analysis are provided in Supplement 1. The resulting clusters of healthier patients (*n* = 28), referred to as group “mild AC,” and sicker patients (*n* = 22), referred to as group “severe AC,” showed comparable group sizes. Mean LVA ± SD was 4.5 ± 2.6 cm^2^ (< 1.0 mV) in *group mild AC*, and 31.5 ± 17.8 cm^2^ (< 1.0 mV) in *group severe AC*. Consistent distribution of NT-proBNP levels between groups (*mild AC*: 125 (48, 189) pg/ml, *severe AC*: 408 (254, 557) pg/ml, *p* < 0.0001) and CHA_2_DS_2_-VASc (*mild AC*: 1 (1, 2), *severe AC*: 3 (3, 4), *p* < 0.0001) served as proof of concept.

### Non-invasive prediction of AC-severity

**Fig. 2:**
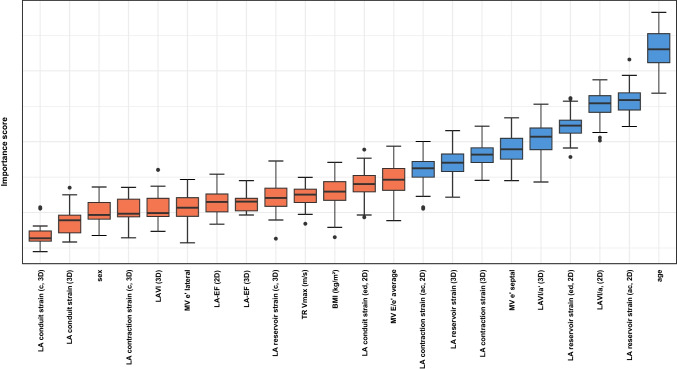
Display of all features evaluated with the Boruta algorithm as a function of the feature importance score. Features that are classified as significant for the prediction model based on their importance scores are colored blue. Features that are classified as nonsignificant for the prediction model are colored orange. 2D (echocardiographic assessment with a 2D data set); 3D (echocardiographic assessment with a 3D data set); ac (zero-strain reference set at atrial contraction); ed (zero-strain reference set a ventricular end-diastole); LA-EF (LA ejection fraction); c (circumferential strain)

**Fig. 3:**
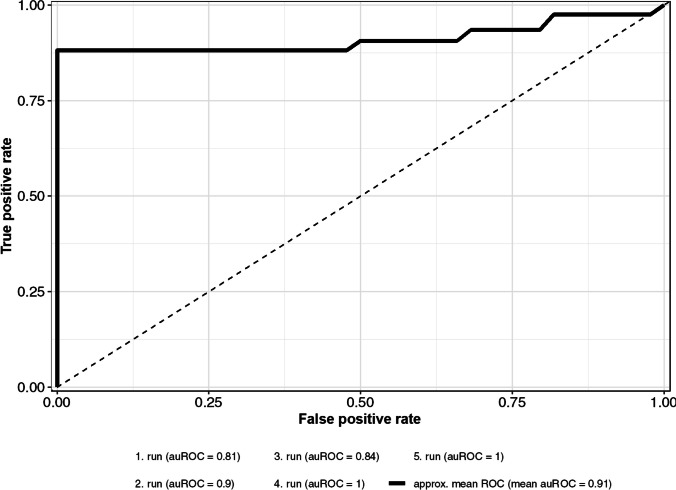
Performance of the support vector machine

We developed a machine learning based approach capable of classifying the severity of AC (*mild* vs *severe*) using easily measurable non-invasive parameters: From an initial set of 22 parameters, 9 features were selected for the subsequent prediction model [14]. Figure 2 displays all the features evaluated with the Boruta algorithm and the ranking of each feature according to its relative importance. The features age, ed LA reservoir strain (2D, 3D), ed LA contraction strain (2D, 3D), LAVI/*a*′ (2D, 3D) as a marker of LA pump function, and the diastolic marker *e*′ measured at the basal LV septum ranked highest in the model’s feature importance. SVM-based classification and assessment of its performance revealed a mean AUROC of 0.9 (95% CI 0.83–0.99) for the reduced non-invasive parameter set. Figure 3 displays the mean ROC and mean AUROC.

### Comparison of echocardiographic and clinical parameters between groups

**Fig. 4:**
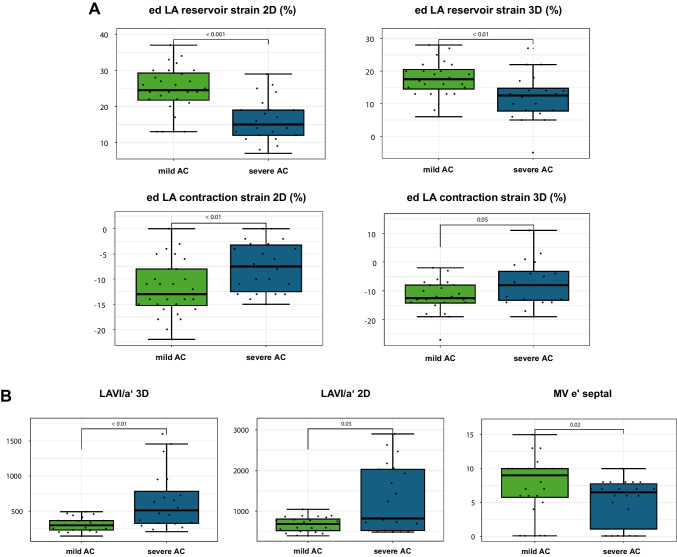
Pairwise comparison of the selected echocardiographic parameters between groups *mild AC* and *severe AC*. **A** LA strain reservoir strain with the zero-strain reference set at end-diastole (ed) and LA contraction strain with the zero-strain reference set at end-diastole (ed) assessed in 2D and 3D. **B** Non-strain-dependent parameters: LA volume index (LAVI)/*a*′ assessed in 2D and 3D and mitral (MV) tissue doppler velocity *e*′ measured at the LV septum

LVEF and LV-GLS were similar in the groups *mild* and *severe AC*. Subjects in group *severe AC* were older (56 (52, 64) years vs. 70 (67, 74) years) and had a higher burden of cardiovascular risk factors (RFs) and comorbidities compared to group *mild AC*: Group *severe AC* showed worse renal function compared to group *mild AC* (glomerular filtration rate 81 vs. 63 ml/min/1.73m^2^), the percentage of persisting AF was nominally higher (77.3 vs 53.6%), and more patients were diabetics. Characteristics of group *mild AC* and group *severe AC* are further detailed in Table 2. All echocardiographic parameters that were selected for the prediction model differed significantly between groups *mild* and *severe AC* as displayed in Table 3. Group *mild AC* consistently showed higher strain values, representing better LA compliance and LA contractility, compared to group *severe AC*: 2D ac LA reservoir strain (22% (19, 25) vs 15% (11, 18)) and 2D ed LA reservoir strain (24.5% (22, 29) vs 15% (12, 19)) (both *p* < 0.001). Quantifying LA contractility, 2D ed LA contraction strain was also higher in group *mild AC* compared to group *severe AC*: 13% (8, 15) vs 7.5% (3, 13), *p* < 0.01. LAVI/*a*′, another parameter for characterization of LA pump function with higher values indicating impaired LA systolic function, was higher in group *severe AC* compared to group *mild AC* measured in 2D: 818 (524, 2029) vs 685 (520, 804), *p* < 0.03 and in 3D: 510 (326, 781) vs 297 (231, 365), *p* < 0.01). Figure 4 visualizes the pairwise comparison of the selected reservoir and contraction strain parameters (Fig. 4A) as well as the metric echocardiographic parameters LAVI/*a*′ and MV *e*′ septal (Fig. 4B) between groups *mild AC* and *severe AC*.

**Table 2:** Characteristics of group *mild AC* and group *severe AC*

	Mild AC *n* = 28	Severe AC *n* = 22	*p*-value
Age (years)	56 (52–64)	70 (67–74)	< 0.001
Male sex, % (*n*)	71.4 (20)	50 (11)	0.25
BMI (kg/m^2^)	28.6 (25.8–31.2)	26.6 (23.7–29.4)	0.13
**Atrial fibrillation**			
CHA2DS2-VASc score	1 (1–2)	3 (3–4)	< 0.001
Paroxysmal AF, % (*n*)	46.4 (13)	22.7 (5)	0.25
Persistent AF, % (*n*)	53.6 (15)	77.3 (17)	0.25
AA medication, % (*n*)	28.6 (8)	50 (11)	0.25
**Cardiovascular risk factors**			
Arterial hypertension, % (*n*)	67.9 (19)	90.9 (20)	0.17
Previous stroke or TIA, % (*n*)	0 (0)	9.1 (2)	0.29
Diabetes mellitus, % (*n*)	7.1 (2)	22.7 (5)	0.3
**Electroanatomical mapping**			
Mapping points per map, *n*	5827 (5214–7169)	5648 (5237–6660)	0.79
LVA cm^2^ (< 1.0 mV)	4.1 (2.2–6.6)	27.2 (16.9–46.1)	< 0.001
LVA cm^2^ (< 0.5 mV)	0.6 (0.2–1.4)	6.9 (4.2–16.9)	< 0.001
**Laboratory values**			
CRP (mg/dl)	0.1 (0.1–0.3)	0.1 (0.1–0.2)	0.8
eGFR (mg/ml/1.73 m^2^)	80.5 (73.5–89.2)	63 (56.5–74.5)	< 0.01
NT-proBNP (pg/ml)	125 (48–189)	408 (254–557)	< 0.001
HbA1c (%)	5.7 (5.5–5.8)	5.8 (5.4–6.0)	0.48
**Echocardiography**			
LVEF biplan (%)	62 (60–64)	60 (56–63)	0.3
GLPS 3D (-%)	17.5 (13.5–20)	16 (14.5–18.5)	0.8
LAVI 2D (ml/m^2^)	29 (26–35)	38 (30–50)	0.04
* E*/*e*′	6.3 (5.4–8.0)	8.9 (6.8–10.4)	0.03
TR Vmax (m/s)	2.4 (2.3–2.7)	2.7 (2.5–3)	0.12
**Outcome**			
AF recurrence, % (*n*)	10.7 (3)	40.9 (9)	< 0.05

Linear trait values are presented as median with interquartile range (25th–75th percentile). Relative and absolute frequencies of categorical variables are presented. *p*-values refer to overall test across subgroups (Fisher’s exact test (dichotomous variables) or Mann–Whitney *U* test (median))

*BMI* body mass index, *AF* atrial fibrillation, *AA* antiarrhythmic, *LVA* low voltage area, *CRP* C-reactive protein, *eGFR* estimated glomerular filtration rate, *NT-proBNP* N-terminal pro-B-type natriuretic peptide, *LVEF* left ventricular ejection fraction, *GLPS* global longitudinal strain, *LAVI* left atrial volume index, *TR Vmax* maximal velocity of tricuspid regurgitation

**Table 3:** Comparison of selected echocardiographic parameters between groups *mild AC* and *severe AC*

	Mild AC	Severe AC	*p*-value
**LA strain**			
2D reservoir strain (R) (%)	24.5 (21.8–29.2)	15 (12–19)	< 0.001
2D reservoir strain (P) (%)	22 (19–25.2)	14.5 (11–17.8)	< 0.001
2D contraction strain (%)	− 13 (−15.2– − 8)	− 7.5 (− 12.5– − 3.2)	< 0.01
3D reservoir strain (R) (%)	17.5 (14.5–20.5)	12.5 (7.8–14.8)	< 0.01
3D contraction strain (R) (%)	− 12.5 (− 14.2– − 8)	− 8 (− 13.2– − 3.2)	0.05
**Non-strain-dependent parameters**			
MV *e*′ sept. (cm/s)	9 (5.8–10)	6.5 (1.1–7.8)	0.02
LAVI/*a*′ 2D	685 (520–804)	818 (524–2029)	0.03
LAVI/*a*′ 3D	297 (231–365)	510 (326–781)	< 0.01

Linear trait values are presented as median with interquartile range (25th–75th percentile). *p*-values refer to Mann–Whitney *U* test (median). R = zero-strain reference set at the ventricular end-diastole. P = zero-strain reference set at the onset of atrial contraction

*LA* left atrial, *LAVI* left atrial volume index

### AF recurrence

Structured telephone interviews and analyses of resting and Holter-ECGs revealed a significantly different rhythm stability between both groups depending on the severity of AC: During the follow-up period ≤ 12 months, AF recurrence rates were significantly lower in group *mild AC* (10.7%, *n* = 3/28) compared to group *severe AC* (40.9%, *n* = 9/22, *p* < 0.05).

## Discussion

The current study investigated the non-invasive prediction of AC severity in a cohort of patients with paroxysmal or persistent AF. The following key findings could be identified: (i) Machine learning–supported algorithms allow non-invasive prediction of groups with mild respectively severe AC. (ii) Besides the factor age, dynamic echocardiographic parameters are most important for non-invasive prediction of the severity of AC. (iii) AC severity, assessed by electro-anatomical mapping, is associated with an increased 12-month AF recurrence after primary PVI.

Previous data underpins that it is feasible to diagnose AC using high-resolution electroanatomical mapping systems and strong evidence exists that early diagnosis of AC may impact stroke risk prevention and AF treatment [7–9, 19–21]. However, as an invasive procedure, LA electro-anatomical mapping is limited to a small, selected number of patients suffering from AF and is not suitable for screening larger populations. Therefore, a compelling need exists to non-invasively predict AC as well as AC severity. We demonstrate that the applied novel machine learning–based approach allows non-invasive prediction of groups with mild respectively severe AC with a promising predictive capacity (mean auROC 0.9).

### Diagnosis of AC derived from high-resolution electroanatomical mapping

There can be no doubt that the invasive detection of significant LVAs within the LA myocardium signifies an altered myocardial structure in the sense of increased fibrosis and reduced density of cardiomyocytes, as it can be expected in AC. This concept is clearly supported by the fact that the association of fibrosis detected in cardiac magnetic resonance imaging (MRI) and invasively detected LVAs has long been established for ventricular myocardium [22–24]. As a result, current ablation strategies for ventricular tachycardias are adapted accordingly [25]. Due to the limitation in spatial resolution, cardiac MRI is not equally helpful to directly detect atrial pathologies [4–6]. Nevertheless, a similar association of LVAs and fibrosis can be assumed. We are confident that LVA from high-resolution maps is a key metric for diagnosing AC. However, it is still unclear, which quantitative values invasively detected LVAs must reach to signify clinically relevant AC. To the best of our knowledge, this study is first to investigate invasively assessed AC avoiding both arbitrary LVA cutoff values for the definition of AC as well as a single, non-evidence-based threshold for demarcation of LA substrate. All studies published to date that assessed the presence of AC using electro-anatomical maps neither methodologically justify the bipolar LA voltage threshold value < 0.5 mV used to indicate low-voltage substrate nor the surface threshold values applied (> 5 cm^2^ resp. ≥ 2 cm^2^ resp. LVA ≥ 3% of LA surface) to define the presence of AC [8–10]. This study challenges the non-evidence-based threshold of < 0.5 mV that is commonly used to define low-voltage substrate. We analyzed two thresholds using both univariate and bivariate approaches and included a higher threshold (< 1.0 mV). The rationale arises from our clinical observation of significant variation in mean voltage among patients. This variation may be explained by differing degrees of atrial myocardial hypertrophy, as hypertrophy is often associated with increased signal amplitudes. Using a threshold of < 0.5 mV may therefore underestimate the severity of AC in individual patients. Our results support the relevance of this strategy and higher thresholds (< 1.0 mV) for delineation of low-voltage substrate. Further, our approach, which assumed a spectrum of different degrees of AC in the investigated cohort, dispensed with a pre-defined cutoff for the diagnosis of AC. Avoiding such arbitrary cutoff values for the definition of AC through the use of unsupervised clustering is a key concept of our methodology. This approach, focusing on the metric LVA, addresses the methodological limitations of prior studies—such as arbitrary cutoffs and/or merging LVA with other variables—early in the analysis. Therefore, confounders were deliberately not considered in the first step of the analysis. The pathophysiological relevance of this unsupervised, LVA-dependent clustering approach is further emphasized by distinct differences between groups *mild* and *severe AC* in clinical parameters and outcomes previously associated with AC [9, 10, 26, 27]. Therefore, the current dataset and the methods applied herein could form the basis for a reliable and reproduceable approach to invasive classification and characterization of AC.

### Spatial resolution of electro-anatomical mapping

As the atrial myocardium is thin and pathologic processes like inflammation and micro-ischemia may initially lead to fine-spotted tissue alterations, it is essential that the spatial resolution achieved during electro-anatomical mapping is of highest quality. For that reason, we here analyzed LA 3D-maps acquired with a multipolar mapping catheter of the newest generation (OCTARAY™, Biosense Webster, Irvine, CA, USA) that was made available ahead of time in the context of a limited market release. As a result, a unique feature of this work constitutes the high number of mapping points acquired for invasive electroanatomical LA assessment. To the best of our knowledge, this study is the first to obtain an average of more than 6500 points (6517 ± 2067 points/map) during electro-anatomical mapping in SR in the context of assessment of AC. The number of mapping points in previous studies assessing LA LVAs ranged from 102 points/map to a maximum of approximately 3000 points/map [7–10, 28, 29]. This is particularly important as the significantly higher spatial resolution provided in this dataset enables a more accurate delineation of LA substrate and additionally allows visualization of LA substrate that was most likely not recorded previously.

### Concepts for non-invasive prediction of AC

To date, a limited number of studies investigated the non-invasive predictability of LVAs signifying AC. There is one study that also evaluated the value of echocardiographic parameters for the prediction of AC [9]. This study showed that LA reservoir strain < 23.5% predicted AC with an AUROC of 0.88 (derivation cohort). In the validation cohort, LA reservoir strain < 23.5% and total LA EF < 34% were associated with AC in 23 of 30 patients undergoing PVI (76%). However, as annotated above, AC was arbitrarily defined at LVA ≥ 2 cm^2^.

In addition, simple non-invasive risk stratification models like “AF-SCORE” and “DR-FLASH” have been proposed to stratify AF patients for the presence of invasively assessed AC without the use of echocardiographic parameters [8, 29]. The “modified APPLE score” integrates basic echocardiographic features in its risk stratification model and applies elements from both approaches mentioned above. However, with an AUROC of 0.79 (AF-SCORE), 0.80 (DR-FLASH), and 0.78 (modified APPLE score), these models only offer moderate discriminative power for the diagnosis of AC [8, 28, 29]. The current study determined a mean AUROC of 0.9, thus exceeding the predictive utility of the studies mentioned above. Importantly, as all other studies used arbitrary thresholds for the definition of AC and as the spatial resolution of electro-anatomical maps was significantly lower, the comparability of the different model’s predictive capacity is methodically limited.

### Echocardiographic parameters characterizing AC

Any severity of AC will induce impairment of the LA function due to the destruction of myocardial integrity. These functional impairments may impact active (contractility) as well as passive features (compliance). Due to significant technological improvements during recent years, a comprehensive assessment of LA function based on echocardiography is possible and easily feasible. Indeed, our model’s algorithm attributed the dynamic echocardiographic factors LA reservoir and LA contraction strain—in addition to the well-established factor age—a high weight for the prediction of AC. This finding stands in line with other studies that have also shown that LA deformation imaging, particularly LA reservoir strain, is an important parameter for the echocardiographic characterization and diagnosis of AC [9, 10, 26]. Studies defining physiological values for respective strain measurements are scarce. A meta-analysis by Pathan et al. reported normal values of 39% for LA reservoir strain and 17% for LA contraction strain derived from a population including more than 2500 healthy subjects [30]. Eichenlaub et al. reported LA reservoir strain < 23.5% and LA conduit strain ≤ 13.4% to be predictive for invasively defined AC [9, 26]. Our distribution of LA ed reservoir resp. conduit resp. contraction strain (15% resp. 8% resp. 7.5% in group *severe AC*, 24.5% resp. 13% resp. 13% in group *mild AC*) correlates with the distribution of LA ed reservoir resp. conduit resp. contraction strain in the above-named study (15% resp. 11% resp. 4% in group AC, 30% resp. 17% resp. 12% in group no AC). The slight differences may be explained by the fact that Eichenlaub et al. included healthier patients in their AC group due to the arbitrary cutoff value for the LVA-derived definition of AC ≥ 2 cm^2^ and both studies analyzed a comparatively small number of patients. The relevance of LA deformation imaging in the context of AF and AC is further emphasized as it demonstrated to predict incident AF [27], arrhythmia recurrence after PVI [9], and risk of thromboembolism [31].

In addition to these data, our study revealed a clear association between LA pump function and AC. Among others, LA pump function can be described by the ratio of LAVI/*a*′. This parameter combines measures of structural und functional LA remodeling. In line with previous data, our study showed a significant difference of LAVI/*a*′ between groups *mild* vs *severe AC* (LAVI/*a*′ 3D: 297 vs 510, *p* < 0.01), and our model’s algorithm assigns LAVI/*a*′ high importance within the parameter selection. The statistical significance and importance of LAVI/*a*′ in the context of AF and AC is further emphasized by the fact that it was shown to predict subclinical AF, an accepted hallmark of AC, in patients with embolic strokes of unknown source (ESUS) (cutoff values LAVI/*a*′ > 282 resp. 367) [32, 33].

### AC and AF recurrence following PVI

Our data show a clear association of AC severity, as assessed by invasive electro-anatomical mapping, and AF recurrence rates ≤ 12 months after primary PVI. Our results are in line with a previous work that also showed increased rates of AF recurrence in patients with larger proportions of LVA [9]. We are convinced that the association of AC severity and outcomes following AF ablation underlines the clinical significance of invasively assessed AC and its potential impact on invasive AF treatment.

## Conclusion

Among patients qualifying for PVI, machine learning analysis of high-resolution LA maps allowed to identify subgroups with mild and severe AC avoiding the use of arbitrary LVA thresholds. These subgroups were predicted non-invasively with good accuracy using a machine learning approach that incorporated a set of echocardiographic markers. This dataset and the methods applied could form a true basis for non-invasive diagnosis and characterization of AC and advance the clinical triage of patients with AF.

## Limitations

No healthy participants without LA disease were examined in the study. Although we examined a broad spectrum of patients, a tendency of the measured parameters towards pathological values cannot be excluded, and no threshold for the definition of a healthy LA can be stated. Further trials investigating patients without LA disease are needed to establish such a threshold value. Due to the limited number of patients, there is a possibility of overfitting despite the careful selection of the machine learning methods and the use of cross-validation. To improve the generalizability of the model, (i) a larger and more diverse patient population and (ii) external validation will be beneficial. This is particularly relevant when comparing the predictive capabilities of different risk stratification models. Further, the number of resting and Holter-ECGs performed ≤ 12 months varied between patients, increasing the susceptibility for error of our outcome analysis.

## Supplementary Information

Below is the link to the electronic supplementary material.

Supplementary file1 (DOCX 71 KB)

## References

[1] Hindricks G, et al. 2020 ESC Guidelines for the diagnosis and management of atrial fibrillation developed in collaboration with the European Association for Cardio-Thoracic Surgery (EACTS): The Task Force for the diagnosis and management of atrial fibrillation of the European Society of Cardiology (ESC) Developed with the special contribution of the European Heart Rhythm Association (EHRA) of the ESC. Eur Heart J. 2021;42(5):373–498. 32860505 10.1093/eurheartj/ehaa612

[2] Goette A, et al. EHRA/HRS/APHRS/SOLAECE expert consensus on atrial cardiomyopathies: definition, characterization, and clinical implication. Heart Rhythm. 2017;14(1):e3–40. 27320515 10.1016/j.hrthm.2016.05.028 PMC5548137

[3] Kreimer F, Gotzmann M. Left atrial cardiomyopathy - a challenging diagnosis. Front Cardiovasc Med. 2022;9:942385. 35845077 10.3389/fcvm.2022.942385 PMC9280085

[4] Bijvoet GP, et al. Correlation between cardiac MRI and voltage mapping in evaluating atrial fibrosis: a systematic review. Radiol Cardiothorac Imaging. 2022;4(5):e220061. 36339060 10.1148/ryct.220061 PMC9627236

[5] Chen J, et al. Extent and spatial distribution of left atrial arrhythmogenic sites, late gadolinium enhancement at magnetic resonance imaging, and low-voltage areas in patients with persistent atrial fibrillation: comparison of imaging vs. electrical parameters of fibrosis and arrhythmogenesis. Europace. 2019;21(10):1484–93. 31280323 10.1093/europace/euz159

[6] Eichenlaub M, et al. Comparison of various late gadolinium enhancement magnetic resonance imaging methods with high-definition voltage and activation mapping for detection of atrial cardiomyopathy. Europace. 2022;24(7):1102–11. 35298612 10.1093/europace/euac010

[7] Muller P, et al. Association of left atrial low-voltage area and thromboembolic risk in patients with atrial fibrillation. Europace. 2018;20(FI_3):f359–65. 29016757 10.1093/europace/eux172

[8] Muller-Edenborn B, et al. Determinants of fibrotic atrial cardiomyopathy in atrial fibrillation. A multicenter observational study of the RETAC (reseau europeen de traitement d’arrhythmies cardiaques)-group. Clin Res Cardiol. 2022;111(9):1018–27. 34854991 10.1007/s00392-021-01973-1 PMC9424172

[9] Eichenlaub M, et al. Echocardiographic diagnosis of atrial cardiomyopathy allows outcome prediction following pulmonary vein isolation. Clin Res Cardiol. 2021;110(11):1770–80. 33914144 10.1007/s00392-021-01850-x PMC8563528

[10] Watanabe Y, et al. Mechanical and substrate abnormalities of the left atrium assessed by 3-dimensional speckle-tracking echocardiography and electroanatomic mapping system in patients with paroxysmal atrial fibrillation. Heart Rhythm. 2015;12(3):490–7. 25485778 10.1016/j.hrthm.2014.12.007

[11] Lang RM, et al. Recommendations for cardiac chamber quantification by echocardiography in adults: an update from the American Society of Echocardiography and the European Association of Cardiovascular Imaging. Eur Heart J Cardiovasc Imaging. 2015;16(3):233–70. 25712077 10.1093/ehjci/jev014

[12] Nagueh SF, et al. Recommendations for the evaluation of left ventricular diastolic function by echocardiography: an update from the American Society of Echocardiography and the European Association of Cardiovascular Imaging. Eur Heart J Cardiovasc Imaging. 2016;17(12):1321–60. 27422899 10.1093/ehjci/jew082

[13] Badano LP, et al. Standardization of left atrial, right ventricular, and right atrial deformation imaging using two-dimensional speckle tracking echocardiography: a consensus document of the EACVI/ASE/Industry Task Force to standardize deformation imaging. Eur Heart J Cardiovasc Imaging. 2018;19(6):591–600. 29596561 10.1093/ehjci/jey042

[14] Kursa MB, Rudnicki WR. Feature selection with the Boruta package. J Stat Softw. 2010;36(11):1–13.

[15] Honaker J, King G, Blackwell M. Amelia II: a program for missing data. J Stat Softw. 2011;45(7):1–47.

[16] Kuhn M. Building predictive models in R using the caret package. J Stat Softw. 2008;28(5):1–26.27774042

[17] Kuhn M, Vaughan D, Hvitfeldt E. Yardstick: tidy characterizations of model performance*.* R package version 1.3.1, 2024.

[18] Benjamini Y. Yekutieli D. The control of the false discovery rate in multiple testing under dependency. Annals Statistics, 2001;29(4):1165–1188, 24.

[19] Yamaguchi T, et al. Atrial structural remodeling in patients with atrial fibrillation is a diffuse fibrotic process: evidence from high-density voltage mapping and atrial biopsy. J Am Heart Assoc. 2022;11(6):e024521. 35261287 10.1161/JAHA.121.024521 PMC9075313

[20] Vlachos K, et al. Low-voltage areas detected by high-density electroanatomical mapping predict recurrence after ablation for paroxysmal atrial fibrillation. J Cardiovasc Electrophysiol. 2017;28(12):1393–402. 28884923 10.1111/jce.13321

[21] Yamaguchi T, et al. Long-term results of pulmonary vein antrum isolation in patients with atrial fibrillation: an analysis in regards to substrates and pulmonary vein reconnections. Europace. 2014;16(4):511–20. 24078342 10.1093/europace/eut265

[22] Marchlinski FE, et al. Linear ablation lesions for control of unmappable ventricular tachycardia in patients with ischemic and nonischemic cardiomyopathy. Circulation. 2000;101(11):1288–96. 10725289 10.1161/01.cir.101.11.1288

[23] Wijnmaalen AP, et al. Head-to-head comparison of contrast-enhanced magnetic resonance imaging and electroanatomical voltage mapping to assess post-infarct scar characteristics in patients with ventricular tachycardias: real-time image integration and reversed registration. Eur Heart J. 2011;32(1):104–14. 20864488 10.1093/eurheartj/ehq345

[24] Kuo L, et al. Multimodality imaging to guide ventricular tachycardia ablation in patients with non-ischaemic cardiomyopathy. Arrhythm Electrophysiol Rev. 2020;8(4):255–64. 32685156 10.15420/aer.2019.37.3 PMC7358957

[25] Tanawuttiwat T, Nazarian S, Calkins H. The role of catheter ablation in the management of ventricular tachycardia. Eur Heart J. 2016;37(7):594–609. 26324538 10.1093/eurheartj/ehv421

[26] Eichenlaub M, et al. Left atrial hypertension, electrical conduction slowing, and mechanical dysfunction - the pathophysiological triad in atrial fibrillation-associated atrial cardiomyopathy. Front Physiol. 2021;12: 670527. 34421634 10.3389/fphys.2021.670527 PMC8375593

[27] Park JJ, et al. Left atrial strain as a predictor of new-onset atrial fibrillation in patients with heart failure. JACC Cardiovasc Imaging. 2020;13(10):2071–81. 32682715 10.1016/j.jcmg.2020.04.031

[28] Seewoster T, et al. Prediction of low-voltage areas using modified APPLE score. Europace. 2021;23(4):575–80. 33279992 10.1093/europace/euaa311

[29] Kosiuk J, et al. Prospective, multicenter validation of a clinical risk score for left atrial arrhythmogenic substrate based on voltage analysis: DR-FLASH score. Heart Rhythm. 2015;12(11):2207–12. 26144350 10.1016/j.hrthm.2015.07.003

[30] Pathan F, et al. Normal ranges of left atrial strain by speckle-tracking echocardiography: a systematic review and meta-analysis. J Am Soc Echocardiogr. 2017;30(1):59-70.e8. 28341032 10.1016/j.echo.2016.09.007

[31] Sade LE, et al. Left atrial mechanics for secondary prevention from embolic stroke of undetermined source. Eur Heart J Cardiovasc Imaging. 2022;23(3):381–91. 33206942 10.1093/ehjci/jeaa311

[32] Stahrenberg R, et al. Transthoracic echocardiography to rule out paroxysmal atrial fibrillation as a cause of stroke or transient ischemic attack. Stroke. 2011;42(12):3643–5. 21998056 10.1161/STROKEAHA.111.632836

[33] Sieweke JT, et al. Septal total atrial conduction time for prediction of atrial fibrillation in embolic stroke of unknown source: a pilot study. Clin Res Cardiol. 2020;109(2):205–14. 31236691 10.1007/s00392-019-01501-2 PMC6989646

