# Supplementary material for: Non-invasive prediction of atrial cardiomyopathy characterized by multipolar high-density contact mapping
Source: J Interv Card Electrophysiol. 2025 Feb 3;68(4):865–76. doi: 10.1007/s10840-025-02001-2 (PMC12246000; doi:10.1007/s10840-025-02001-2)
Supplement: Supplementary file 1 — Supplementary file1 (DOCX 71 KB) [file 10840_2025_2001_MOESM1_ESM.docx]

**Supplement 1**

Clustering was performed in an unsupervised manner, both in determining the number of clusters and the assignment of clusters. We performed different computational experiments using both univariate and bivariate input variables. For the univariate input variables, we separately used the two different voltage thresholds <0.5 mV and <1.0 mV. For the bivariate input variable, we used a two-dimensional vector containing the low-voltage area (LVA) values for the thresholds <0.5 mV and <1.0 mV.

The number of clusters was determined in an unsupervised manner by calculating the Bayesian information criteria (BIC) score for different numbers of clusters, ranging from one to nine (provided that the model could be estimated). The optimal number of clusters was selected on the highest BIC score. Following this approach, we identified the following numbers of clusters:

i) for univariate input LVA <0.5 mV: four,

ii) for univariate input LVA <1.0 mV: two, and

iii) for bivariate input: three clusters.

Given the limited amount of data points in our study, we pragmatically selected the input variable and the corresponding number of clusters. For the univariate input of LVA <0.5 mV, the number of clusters (four) was too high, resulting in very small group sizes (8, 10, 11, 21 data points), which could lead to an overfitting in the subsequent analysis of echocardiologic parameters involving the support vector machine (SVM). For the univariate input of LVA <1.0 mV, two clusters were identified with cluster borders in close proximity. The cluster representing severe atrial cardiomyopathy (AC) exhibited relatively high variance, increasing the likelihood that patients with LVA values intermediate between mild and severe AC would be assigned to the severe AC cluster. This observation suggests a relatively conservative assignment to cluster a data point as a patient suffering from severe AC rather than from mild AC. When combining the LVA thresholds and performing clustering on the multivariate input, we identified a third cluster that captured an intermediate cluster between mild and severe AC. However, due to the limited number of data points (n = 50) and the high number of parameters required for the analysis (each cluster is characterized by a 2D vector for the mean values and a covariance matrix with 4 elements, leading to a total of 18 parameters for three clusters), we were cautious about the generalizability of these results.

**Figure:** Left column shows the BIC score depending on the number of clusters. BIC scores could only be calculated if a model with as many clusters could be estimated. Right column shows the cluster assignments of each data point based on the model with the highest BIC score.
